# Stable, synthetic analogs of diadenosine tetraphosphate inhibit rat and human P2X3 receptors and inflammatory pain

**DOI:** 10.1177/1744806916637704

**Published:** 2016-03-29

**Authors:** Viacheslav Viatchenko-Karpinski, Natalia Novosolova, Yevheniia Ishchenko, M Ameruddin Azhar, Michael Wright, Vera Tsintsadze, Ahmed Kamal, Nail Burnashev, Andrew D Miller, Nana Voitenko, Rashid Giniatullin, Natalia Lozovaya

**Affiliations:** 1 Laboratory of Sensory Signaling, Bogomoletz Institute of Physiology, Kiev, Ukraine; 2 International Center for Molecular Physiology, Kiev, Ukraine; 3 Department of Neurobiology, A. I. Virtanen Institute, Kuopio, Finland; 4 Indian Institute of Chemical Technology, Hyderabad, India; 5 Imperial College Genetic Therapies Centre, Department of Chemistry, Imperial College London, London, UK; 6 Institute of Pharmaceutical Science, King’s College London, London, UK; 7 INSERM UMR901 Aix-Marseille Université, Marseille, France; 8 INMED, Institut de Neurobiologie de la Méditerranée, Marseille, France; 9 GlobalAcorn Ltd, London, UK; 10 Kazan Federal University, Kazan, Russia; 11 Neurochlore, Marseille, France

**Keywords:** P2X3 receptors, high-affinity desensitization, stable synthetic diadenosine tetraphosphate analogs, inflammatory pain, dorsal root ganglion

## Abstract

**Background:**

A growing body of evidence suggests that ATP-gated P2X3 receptors (P2X3Rs) are implicated in chronic pain. We address the possibility that stable, synthetic analogs of diadenosine tetraphosphate (Ap_4_A) might induce antinociceptive effects by inhibiting P2X3Rs in peripheral sensory neurons.

**Results:**

The effects of two stable, synthetic Ap_4_A analogs (AppNHppA and AppCH_2_ppA) are studied firstly in vitro on HEK293 cells expressing recombinant rat P2XRs (P2X2Rs, P2X3Rs, P2X4Rs, and P2X7Rs) and then using native rat brain cells (cultured trigeminal, nodose, or dorsal root ganglion neurons). Thereafter, the action of these stable, synthetic Ap_4_A analogs on inflammatory pain and thermal hyperalgesia is studied through the measurement of antinociceptive effects in formalin and Hargreaves plantar tests in rats in vivo. In vitro inhibition of rat P2X3Rs (not P2X2Rs, P2X4Rs nor P2X7Rs) is shown to take place mediated by high-affinity desensitization (at low concentrations; IC_50_ values 100–250 nM) giving way to only weak partial agonism at much higher concentrations (EC_50_ values ≥ 10 µM). Similar inhibitory activity is observed with human recombinant P2X3Rs. The inhibitory effects of AppNHppA on nodose, dorsal root, and trigeminal neuron whole cell currents suggest that stable, synthetic Ap_4_A analogs inhibit homomeric P2X3Rs in preference to heteromeric P2X2/3Rs. Both Ap_4_A analogs mediate clear inhibition of pain responses in both in vivo inflammation models.

**Conclusions:**

Stable, synthetic Ap_4_A analogs (AppNHppA and AppCH_2_ppA) being weak partial agonist provoke potent high-affinity desensitization-mediated inhibition of homomeric P2X3Rs at low concentrations. Therefore, both analogs demonstrate clear potential as potent analgesic agents for use in the management of chronic pain associated with heightened P2X3R activation.

## Introduction

Chronic pain syndrome occurs and persists in a heterogeneous group of etiologically different diseases. It is classified into nociceptive, inflammatory, and neuropathic pain; however, a comorbid mix of both components frequently occurs in postsurgical, osteoarthritic, cancer-related, and back pain. Nociceptive inflammatory pain, in particular, is caused by tissue damage and subsequent release of inflammatory mediators, which leads to the sensitization of peripheral nociceptors.^1,2^ Despite intensive exploration, few treatments of chronic nociceptive pain are known to be effective. ATP-gated P2X3 (P2X3R) and heteromeric P2X2/3 receptors expressed in nociceptive neurons are involved in various chronic pain states.^3–5^ P2X3Rs substantially contribute toward the transmission of pain signals^6–13^ and, therefore, appear to be an appropriate therapeutic target for the treatment of chronic nociceptive pain. The key role of P2X3Rs in the transmission of nociceptive signals has been confirmed by the knockout of P2X2R and P2X3R^8,14,15^ genes and by means of siRNA^16^ or antisense oligonucleotide-mediated gene knockdown.^17,18^

Critically, P2X3R antagonists have analgesic effects in nociceptive and neuropathic pain models.^11,12,19–21^ Indeed several novel nonnucleotide antagonists have been shown to inhibit the activities of P2X3Rs in nociceptive or neuropathic pain.^22–28^ However, despite significant advances in the exploration of P2X3R pharmacology, potent P2X3R-selective antagonists/inhibitors are in relatively short supply. To date, only one major P2X3R-selective antagonist (AF219) has been evaluated in clinic for the relief of pain.^23^ In seeking alternatives, the unusual gating of homomeric P2X3Rs opens up new possibilities for inhibition. Of particular relevance, P2X3Rs exhibit fast desensitization (in the millisecond range) with a very slow recovery (several minutes).^29–31^ Indeed, some agonists such as ATP readily induce use-dependent high-affinity desensitization (HAD) of P2X3Rs.^32,33^ Accordingly, were a ligand to be discovered that was able to induce HAD while functioning only as a weak agonist, then such a ligand could be a powerful pharmacological tool for P2X3R inhibition and a potentially powerful pharmaceutical tool for the inhibition of pain effects. It is in this context that we have been examining the effects of stable, synthetic diadenosine polyphosphate analogs on pain, specifically with reference to P2X3Rs.

Diadenosine polyphosphates (Ap*_n_*As; where *n* is 2–7) are naturally occurring purinergic ligands consisting of two adenosine moieties bridged by a chain of two or more phosphate residues attached at the 5′-position of each ribose ring.^34^ In particular, *P*^1^,*P*^4^-diadenosine tetraphosphate (Ap_4_A) and *P*^1^,*P*^5^-diadenosine pentaphosphate (Ap_5_A) are present in high concentrations endogenously in the secretory granules of chromaffin cells^34^ and in rat brain synaptic terminals.^35^ Upon depolarization, Ap*_n_*As are released in a Ca^2+^-dependent manner^34^ and their potential role as neurotransmitters has been proposed.^35–37^ Ap*_n_*As are known as agonists of some P2XRs and P2Yrs.^38–40^ Ap_4_A and Ap_5_A have also been shown to induce potent desensitization of recombinant P2X3Rs.^41^ Unfortunately, the pharmaceutical and therapeutic potential of Ap*_n_*As is limited by the fact that Ap*_n_*As undergo specific enzymatic cleavage and also nonspecific hydrolytic breakdown in vivo. Fortunately, this in vivo lability issue can be overcome by using synthetic methods to replace one or more of the oxo-bridges in a polyphosphate chain with either aza- or carba-bridges. Here, we report on the effects of using two stable, synthetic Ap_4_A analogs—AppCH_2_ppA (diadenosine 5′,5′′′-*P*^1^,*P*^4^-(β,γ-methylene)tetraphosphate) and AppNHppA (diadenosine 5′,5′′′-*P*^1^,*P*^4^-(β,γ-imido)tetraphosphate)—in a range of in vitro studies designed to understand the mechanism of action and efficacy of these Ap_4_A analogs for the control and management of nociceptive pain responses. Here we show that both induce potent, use-dependent HAD of P2X3Rs (strong antinociceptive activity), while in contrast both are found to be weak, partial P2X3R agonists (weak pronociceptive activity). In addition, we show that both analogs are able to exert potent antinociceptive activities in in vivo animal models of inflammatory pain. Therefore, both could indeed be very powerful pharmaceutical agents for P2X3R inhibition and for the inhibition of nociceptive pain effects.

## Methods

### Ap_n_A analog syntheses

AppNHppA and AppCH_2_ppA were prepared using LysU-mediated synthetic–biosynthetic (chemo-enzymatic) procedures as described previously^42,43^ with rigorous purification by high-performance liquid chromatography.^44,45^

### Cell cultures and transfection

Rat trigeminal, nodose, or dorsal root ganglion (TG, NG, and DRG, respectivley) neurons in culture were prepared as described previously.^46,47^ Neurons were plated on poly-l-lysine (0.2 mg/ml)-coated Petri dishes and cultured for one to two days under an atmosphere containing 5% CO_2_. Cells were used within two days of plating when they lacked processes. HEK293T cells were prepared as reported previously^32,48^ and transfected with rat or human full-length P2X3 cDNA subcloned into pIRES2-EGFP (Clontech, Mountain View, CA, USA).

### Electrophysiological recordings

TG, NG, DRG, or HEK cells were recorded in the whole-cell configuration while being continuously superfused (at 2 ml/min) with control solution containing (in mM): 152 NaCl, 5 KCl, 1 MgCl_2_, 2 CaCl_2_, 10 glucose, and 10 HEPES; pH was adjusted to 7.4 with NaOH and osmolarity was adjusted to 320 mOsM with glucose. Patch pipettes had a resistance of 3 to 4 MΩ when filled with an intracellular equivalent solution containing (in mM): 130 CsCl, 0.5 CaCl_2_, 5 MgCl_2_, 5 K_2_ATP, 0.5 NaGTP, 10 HEPES, and 5 EGTA; pH was adjusted to 7.2 with CsOH. Responses to selective P2X3R agonist α,β-methylene-ATP (α,β-meATP; resistant to ectoATPase hydrolysis, Sigma-Aldrich) were measured using an EPC-9 amplifier operated by HEKA Patch Master software (HEKA Electronik, Germany). Cells were voltage-clamped at −70 mV. In most cells, series resistance was compensated by 80%. To determine EC_50_ values, dose-response curves were constructed for α,β-meATP by administering different agonist doses same cells and fitting data to a logistic equation (Origin 8.0, Microcal, Northampton, MA).

### Drug delivery

Agonists and antagonists were applied (usually for 2 s) via a rapid superfusion system (Rapid Solution Changer RSC-200; BioLogic Science Instruments, Grenoble, France) placed 100 to 150 µm near the cell. The time for the solution exchange across the cell was approximately 30 ms, as judged with liquid junction potential measurements. All chemicals, including enzymes for cell culture, were from Sigma-Aldrich (St. Louis, MO). Culture media were obtained from Invitrogen (Milan, Italy).

### Measurement of nociceptive behavior in formalin test

All animal experiments were carried out in accordance with the “Guide for the Care and Use of Laboratory Animals” as adopted by the Bogomoletz Institute of Physiology Clinic of Animal Care and Use. The formalin test experiments were performed on 21-day-old male Wistar rats, weighing 40 ± 5 g according to the method described previously.^49,50^ Briefly, animal was acclimated to an acrylic observation chamber for at least 1 h before the injection of formalin (50 µl of 0.5% solution in 0.9% saline) into the dorsal surface of the right hindpaw. Immediately after the injection, each animal was returned to the observation chamber and its behavioral characteristics were recorded for 45 min. We noticed consistently observed type of response: spontaneous jerking of the injected paw. The number of jerks was measured for each 1-min block, using self-developed computer software.

### Measurement of hindpaw thermal hyperalgesia

To induce chronic inflammatory hyperalgesia, rats were injected with complete Freund’s adjuvant (CFA, 100 μl of 50% solution) into the plantar surface of the right hindpaw 24 h prior to testing. Control animals were injected with 100 μl of pure saline. Injections of AppCH_2_ppA and AppNHppA (100 nM–100 μM) in 100 μl of 0.9% saline were made just before the beginning of the plantar test.

For intrathecal injection, a polyethylene-10 catheter was inserted into the rat’s subarachnoid space through a small incision at the cisterna magna, and the tip of the catheter was implanted at the L4 spinal segmental level. Animals were allowed to recover for at least four days before behavior tests. AppCH_2_ppA or vehicle (saline) was injected intrathecally (10 µl) and flushed with 10 µl of saline.

The Hargreaves technique^51^ was used for thermal hyperalgesia studies (using the Ugo Basile Model 7370 Plantar Test). Paw withdrawal responses to noxious heat stimuli were measured in rats as described.^52^ To measure response to noxious heat stimuli, each animal was placed in a Plexiglas chamber on a glass plate located above a light box. Radiant heat was applied by focused infrared (IR) beam to the middle of the plantar surface of rat hindpaw. When the animal lifted its foot, the IR beam was turned off. The time interval between the start of the IR beam and the foot lift was defined as the paw withdrawal latency. Each trial was repeated 13 times at 20-min intervals for each paw. A cut-off time of 30 s was used to prevent paw tissue damage.

### Data analysis

The peak amplitudes of the responses were measured using HEKA Patch Master software. For each agonist, dose-response plots were constructed by normalizing data with respect to the maximum response. All data are presented as mean ± SEM (*n* = the number of cells) with statistical significance assessed by paired *t*-test (for parametric data) or Mann-Whitney rank-sum test (for nonparametric data). Best fits of data with a sigmoid function were compared with respective control fits using Origin 8.0 software. A value of *p* < 0.05 was accepted as indicative of statistically significant difference.

## Results

### Inhibitory effects and weak agonist effects of stable Ap_4_A analogs acting on recombinant rat homomeric P2X3 receptors

**Figure 1.**
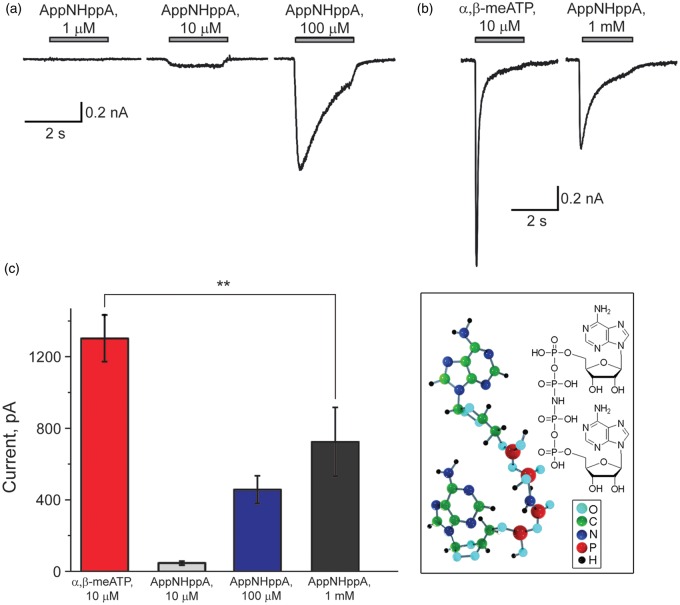
Partial agonist activity of AppNHppA at rat homomeric P2X3 receptors. (a): Example of responses induced by different concentrations of AppNHppA (1 µM–100 µM) applied for 2 s to HEK cells expressing rat P2X3 receptors. (b) Comparison of currents induced by the full agonist of P2X3 receptors α,β-meATP (10 µM, left) and saturating (1 mM, right) concentration of AppNHppA in the same cell. Note significantly smaller amplitude of the latter. (c) Bar graphs showing mean ± SEM amplitude of currents induced by α,β-meATP at 10 µM and AppNHppA at different concentrations (10 µM–1 mM), *n* = 7. Inset: molecular structure of AppNHppA. Here and below *p < 0.05, **p < 0.001, and ***p < 0.0001.

Previous publications have demonstrated that several diadenosine polyphosphates (such as Ap_4_A and Ap_5_A) might be full agonists of rat P2X3R.^40,41^ In our experiments using HEK293 cells expressing rat homomeric P2X3R, the stable, synthetic analog AppNHppA was found not to induce membrane currents at concentrations of 0.1 or 1 µM. However, small and slowly desensitizing currents were generated by 10 or 100 µM AppNHppA (Figure 1(a)). To test whether AppNHppA is a full or partial agonist of P2X3Rs, we compared AppNHppA-induced responses with those induced by the full P2X3R agonist α,β-meATP (Figure 1(b)). In the same cell, we observed that the current response elicited by AppNHppA even at a saturating 1 mM concentration was 41.2 ± 13% (*n* = 3, *p* = 0.009 by paired *t*-test) of the response induced by 10 µM of α,β-meATP (Figure 1(c)). These results indicate that AppNHppA is only a partial agonist of homomeric P2X3Rs and not a full agonist such as Ap_4_A itself.^40,41^

**Figure 2.**
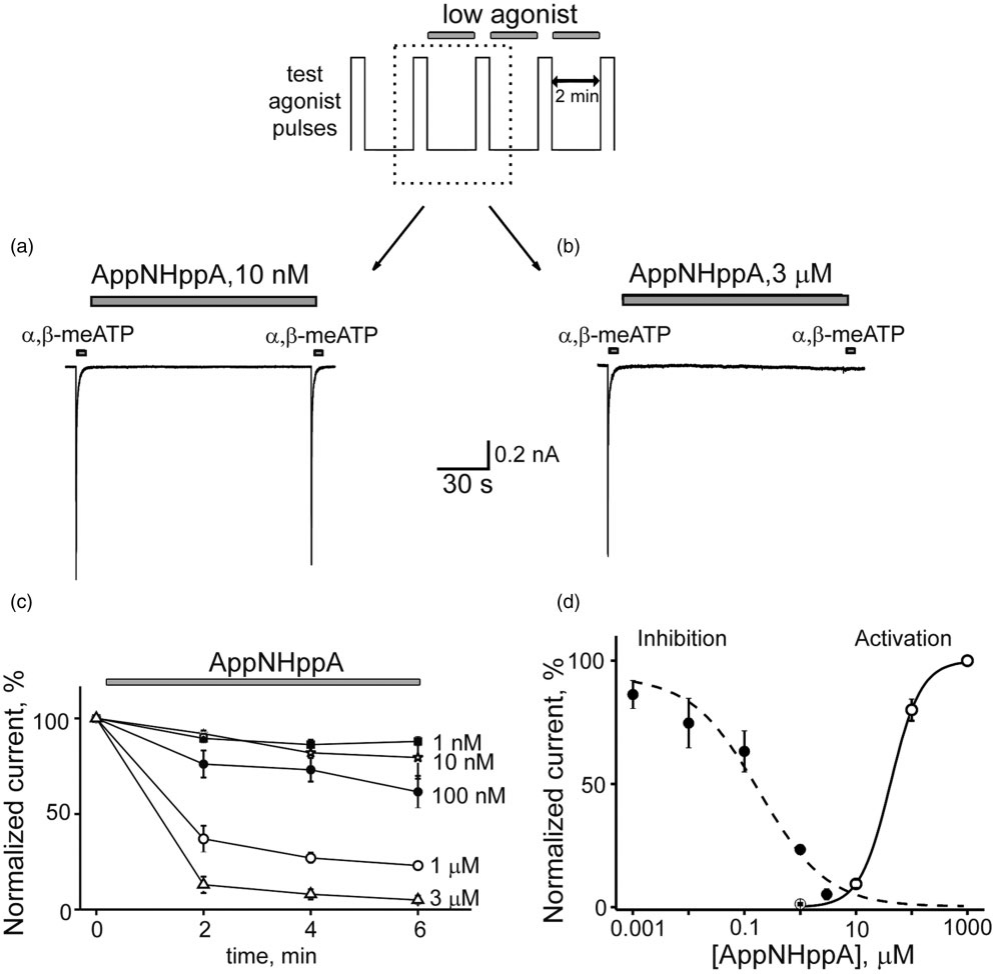
Activation and inhibition of rat P2X3 receptors by AppNHppA. Top: Experimental protocol. Test pulses of 10 µM of α,β-meATP were delivered every 2 min. After two control pulses, AppNHppA at different concentrations was applied for 6 min. Example traces of P2X3R-mediated currents in control and after 2 min application of 10 nM (a) and 3 μM (b) of AppNHppA (corresponding to the part of the experimental protocol outlined by a dotted box). (c) The time course of the current inhibition by AppNHppA at different (1 nM–3 μM) concentrations (*n* = 4–6). Current amplitudes are normalized to control. (d) Dose-response curves for the activation and inhibition of P2X3-mediated currents by AppNHppA.

**Figure 3.**
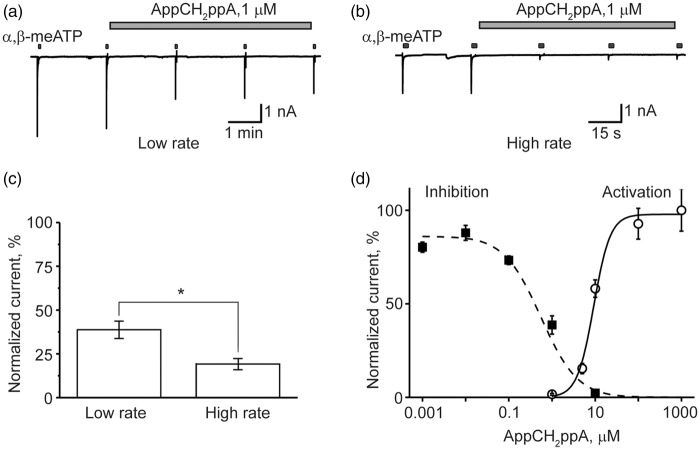
Inhibitory and agonist effect of AppCH_2_ppA on homomeric P2X3 receptors. (a) Example traces of P2X3R-mediated currents in control and after application of 1 µM AppCH_2_ppA. Experimental protocol is the same as shown in Figure 2. Test 10 µM α,β-meATP pulses were applied every 2 min (low rate). (b) The same as in (a), but test α,β-meATP pulses were applied every 30 s (high rate). (c) Bar graphs showing inhibitory action of AppCH_2_ppA at low and high rate of activation. (d) Dose-response curves for the activation and inhibition of P2X3-mediated currents by AppCH_2_ppA.

At subthreshold concentrations, certain P2X3R agonists can inhibit receptor function via the mechanism of HAD.^32,33,53^ Therefore, the inhibitory potency of AppNHppA against P2X3R was evaluated for the potential existence of this HAD effect. To examine for this, repetitive 2-s-long activations of P2X3R (2-min interpulse interval) were induced using α,β-meATP (10 µM) as the full agonist (later on test-pulse) before and after AppNHppA administration (for protocol, see Figure 2 top). When 10 nM of AppNHppA was applied after a control test response to α,β-meATP, the amplitude of the following response was almost unchanged (Figure 2(a)). However, when 3 µM of AppNHppA was administered, a complete inhibition of test responses was induced indicative of the HAD effect (Figure 2(b)). Notably, AppNHppA administered at a concentration below the activation threshold (100 nM; see Figure 2(d)) induced a strong HAD effect on test responses, reducing them by 38.2 ± 8.5 % (*n* = 7, *p* = 0.002). The time course for these HAD inhibitory effects of AppNHppA is shown in Figure 2(c), and the concentration dependence measured at condition approaching saturation (6 min after drug application) suggests an IC_50_ value of 0.20 ± 0.04 µM (*n* = 3–6) (Figure 2(d)). Importantly, the HAD inhibitory curve for AppNHppA did not overlap with the partial agonist P2X3R-activation curve, since the partial agonist effects of AppNHppA on P2X3Rs appeared to require much higher concentrations (EC_50_ = 41.6 ± 1.3 µM; *n*_H_ = 1.58 ± 0.05; *n* = 8; Figure 2(d)). Thus, AppNHppA exhibits a unique profile of activity with a high EC_50_ of 41 µM and low IC_50_ of 200 nM. Should we use the EC_50_/IC_50_ ratio as an indication of activation/inhibition or as an safety/efficiency, then the ratio for AppNHppA is 205, which is ∼20 times higher than corresponding index values for ATP (15.13) and endogenous Ap_4_A (11.48) (relevant EC_50_ and IC_50_ values for ATP and Ap_4_A were taken from literature^41^). For comparison, the HAD inhibitory effects mediated by stable, synthetic Ap_4_A analog AppCH_2_ppA were found similar to those induced by AppNHppA (IC_50_ = 0.55 ± 0.2 µM, *n* = 4–7; Figure 3(a) and (d)), although the partial agonist effect was apparently stronger (EC_50_ = 9.30 ± 1.7 µM; *n*_H_ = 2.27 ± 0.56; n = 5; Figure 3(d)).

To understand the nature of the inhibitory action of Ap_4_A analogs, we applied AppCH_2_ppA to cells stimulated with test pulses either every 2 min or 30 s. Interestingly, with an increased frequency of agonist applications (30 s intervals; Figure 3(b) and (c)), when the fraction of P2X3R in desensitized state was enhanced, the inhibitory activity of AppCH_2_ppA was significantly increased (depression by 81 ± 3 % vs 61.3 ± 5 % at low rate, *p* = 0.014, *n* = 7), suggesting that a depressant effect on P2X3R is use-dependent, via the HAD mechanism.

**Figure 4.**
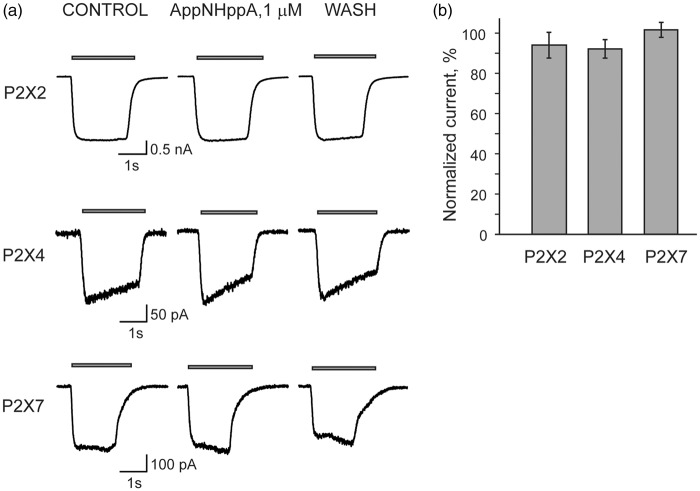
AppNHppA does not inhibit homomeric P2X2, P2X4, and P2X7 receptors. (a) Representative traces of responses induced by 10 µM of ATP applied for 2 s every 2 min to HEK293 cells expressing rat P2X2, P2X4, and P2X7 receptors in control, in the presence, and after wash out of AppNHppA (1 µM). (b) Bar graphs of the effects of AppNHppA on the amplitude of P2X2, P2X4, and P2X7 receptor-mediated currents.

**Figure 5.**
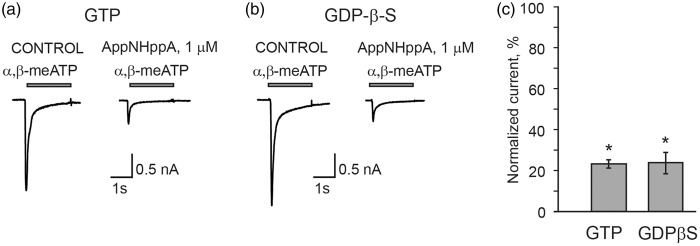
Effect of AppNHppA on P2X3 is not altered in the presence of G-protein inhibitor. Example traces of P2X3-mediated currents in control and after 6 min application of 1 µM AppNHppA with GTP (500 µM, (a)) or GDP-β-S (500 µM, (b)) in the intracellular solution. (c) Bar graph showing the inhibitory action of AppNHppA on P2X3 receptors with GTP and GDP-β-S (23.41 ± 2.01%, *n* = 4, *p* < 0.05 for GTP and 23.93 ± 5.24, *n* = 5, *p* = 0.007 for GDP-β-S).

In contrast, in separate experiments with recombinant P2X2Rs, P2X4Rs, or P2X7Rs expressed in HEK 293 cells, the application of AppNHppA did not produce any HAD or partial agonism effects on any of these receptor-mediated currents (Figure 4). Altogether these data indicate that both stable, synthetic Ap_4_A analogs appear to induce selective inhibition of recombinant P2X3Rs by use-dependent HAD, and exercise partial agonism at best at substantially higher, nonoverlapping concentrations. To test for the possible involvement of metabotropic G-protein coupled P2Y receptors in the observed phenomena of P2X3R inhibition by Ap_4_A analogs, we performed experiments using a nonhydrolyzable analog of GDP and competitive inhibitor of G-proteins, GDP-β-S, (500 µM) introduced into cells. Under these conditions, the inhibitory action of AppNHppA remained unchanged (Figure 5). These results clearly rule out the involvement of P2YRs.

### Modulation of native P2X3 receptors by Ap_4_A analogs in rat cultured sensory neurons

**Figure 6.**
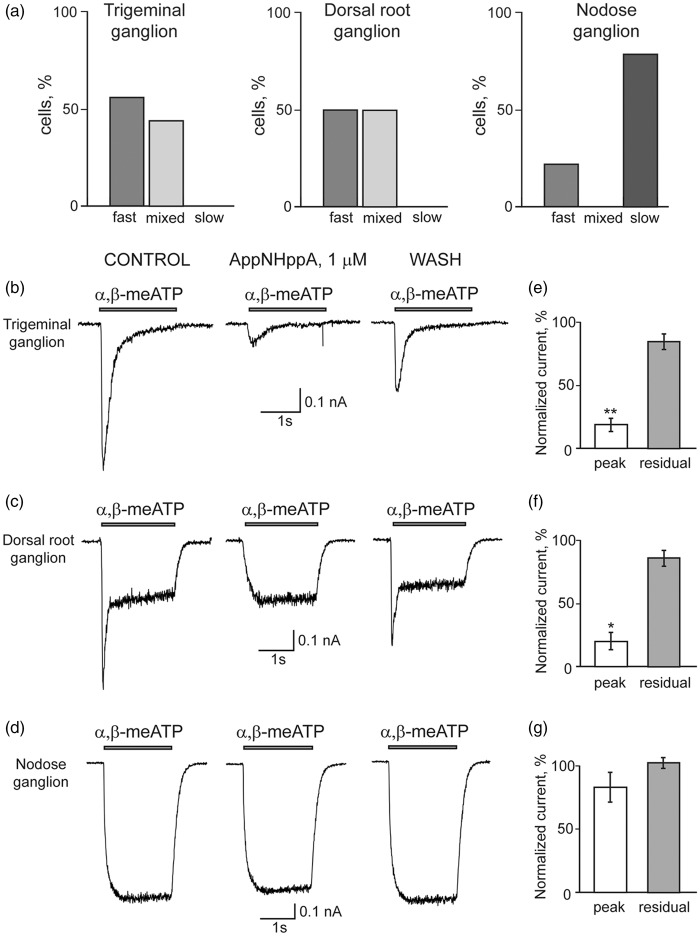
Selective inhibition of P2X3 subunit containing receptors by AppNHppA in rat sensory neurons. (a) The relative proportion of cells with fast, mixed and slow current types elicited by α,β-meATP in trigeminal (TG), dorsal root (DRG), or nodose (NG) neurons. Typical examples of fast, mixed, and slow currents are shown in panels (b) to (d), respectively. Examples showing inhibitory action of AppNHppA (1 µM, 6 min application) on responses evoked by 10 µM α,β-meATP in TG (b), DRG (c), and NG (d) ganglia neurons, respectively. Note preferential inhibition of fast currents. Bar graphs showing the inhibitory action of AppNHppA on fast (peak) and slow (residual, at the end of α,β-meATP application) currents in different ganglia in TG (e), DRG (f), or NG (g) neurons.

Next the effects of AppNHppA on native P2X3Rs in TG, DRG, and NG neurons were investigated. Decay kinetics of ATP response in situ strongly depends on differential contributions from P2X2R and P2X3R subunits.^46,54^ Fast desensitizing (fast) responses (within 1 s from agonist application) correlate with contributions of P2X3R subunits only. Slow desensitizing (sustained) responses (1 to 2 s from agonist application) correlate with contributions of heteromeric P2X2R with P2X3R subunit combinations (P2X2/3Rs).^12,15^ Notably, only homomeric P2X3Rs and heteromeric P2X2/3Rs are activated by 10 µM of α,β-meATP. Following administration of test pulses of α,β-meATP, responses induced in TG neurons were found to be fast (56% of the cells) and mixed (composite of fast and slow desensitizing responses) (44% of the cells) but without slow-type responses (0% of the cells, *n* = 18 cells) (Figure 6(a)). In DRG neurons (*n* = 8), responses induced were 50% fast, 50% mixed, and 0% slow, whereas in NG neurons (*n* = 11), responses induced were 22% fast, 0% mixed, and 78% slow (Figure 6(a)). These data indicate that TG neurons and DRG cells predominantly express homomeric P2X3Rs, while NG neurons express heteromeric P2X2/3Rs subunits. Importantly, administration of AppNHppA (1 µM) selectively inhibited fast responses (Figure 6(b) and (c)), with little effect on mixed or slow responses (Figure 6(d)). Indeed, inhibition of peak currents was observed to be 80.3 ± 4.4% (n = 9, P = 0.0004) in TG neurons, 79.2 ± 5.6% (*n* = 6, *p* = 0.02) in DRG neurons and only 16.8 ± 11.8% (*n* = 9, *p* > 0.05) in NG neurons (Figure 6(e) to (g)). Furthermore, sustained residual components were less inhibited (15 ± 6%, 18 ± 6%, and 2 ± 4% for TG, DRG, and NG neurons, respectively; Figure 6(e) to (g)). Accordingly, these data interlock to support the view that inhibition of cellular responses by AppNHppA is essentially mediated through homomeric P2X3Rs alone and not heteromeric P2X2/3Rs. These data are also consistent with the fact that AppNHppA is also unable to elicit any responses from recombinant P2X2Rs (see earlier). Furthermore, these data obtained with native neurons are completely consistent with our results from studies with recombinant homomeric P2X3Rs (Figures 2 and 3).

### Inhibition of human P2X3 receptors by Ap_4_A analogs

**Figure 7.**
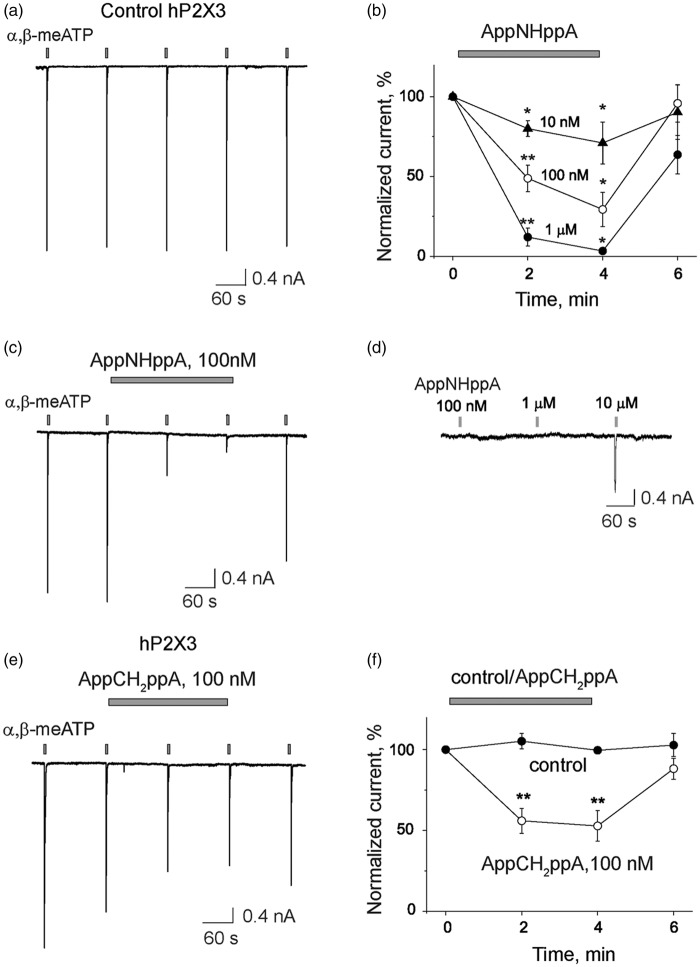
Inhibitory action of AppNHppA on human P2X3 receptors. (a) Representative current responses induced by 2 s applications of α,β-meATP (α,β-ATP) (10 μM) in control; (b) Example of the inhibitory action of AppNHppA (100 nM) on α,β-meATP-induced currents; (c) Dose-dependent depressant action of AppNHppA on α,β-meATP-induced currents (*n* = 3–6, ean ± SEM, *p < 0.05); (d) Representative current responses activated by raising concentrations of AppNHppA. AppNHppA was unable to induce membrane currents at concentrations that resulted in hP2X3R inhibition (threshold concentration for activation was found to be 10 µM AppNHppA); (e) Representative responses showing the inhibitory action of 100 nM AppCH_2_ppA on α,β-meATP-induced currents; (f) Plots showing the depressant action of AppCH_2_ppA on α,β-meATP-induced currents (*n* = 6, mean ± SEM, **p < 0.01).

Next we tested the inhibitory action of AppNHppA and AppCH2ppA in recombinant human P2X3Rs (hP2X3Rs) expressed in HEK293 cells. The inhibitory potency of AppNHppA on hP2X3Rs was found as strong as with rat P2X3Rs (Figure 7). Notably, even 10 nM AppNHppA significantly reduced currents activated by the P2X3R agonist α,β-meATP to 80 ± 5% (*n* = 4, *p* < 0.01 by paired *t-*test) of control values, whereas 100 nM AppNHppA inhibited test responses to 48 ± 8% of control (*n* = 6, *p* < 0.01); and 1 µM AppNHppA inhibited test currents to 12 ± 4% of control (*n* = 3, *p* < 0.01) (Figure 7(b) and (c)). Interestingly, the activation of the currents by AppNHppA (partial agonist activity) was observed only in concentrations exceeding 10 µM similar to results obtained with rat P2X3Rs (Figure 2(d)), in contrast to inhibitory activities that were observed at concentrations 10^3^ lower, once again demonstrating the very low agonist activity of this compound. Likewise, the inhibitory effect of AppCH_2_ppA on hP2X3Rs at the subthreshold concentration of 100 nM (56 ± 7%, *n* = 6, *p* < 0.01) (Figure 7(d) and (e)) was also as strong as on rat P2X3Rs (Figure 3(d)). Notably, responses recovered from inhibition after washout of the both inhibitory agents. No depression was observed in control experiments with the same regular applications of α,β-meATP but without adding Ap_4_A analogs (Figure 7).

### Antinociceptive effects of stable, synthetic Ap_4_A analogs in vivo

**Figure 8.**
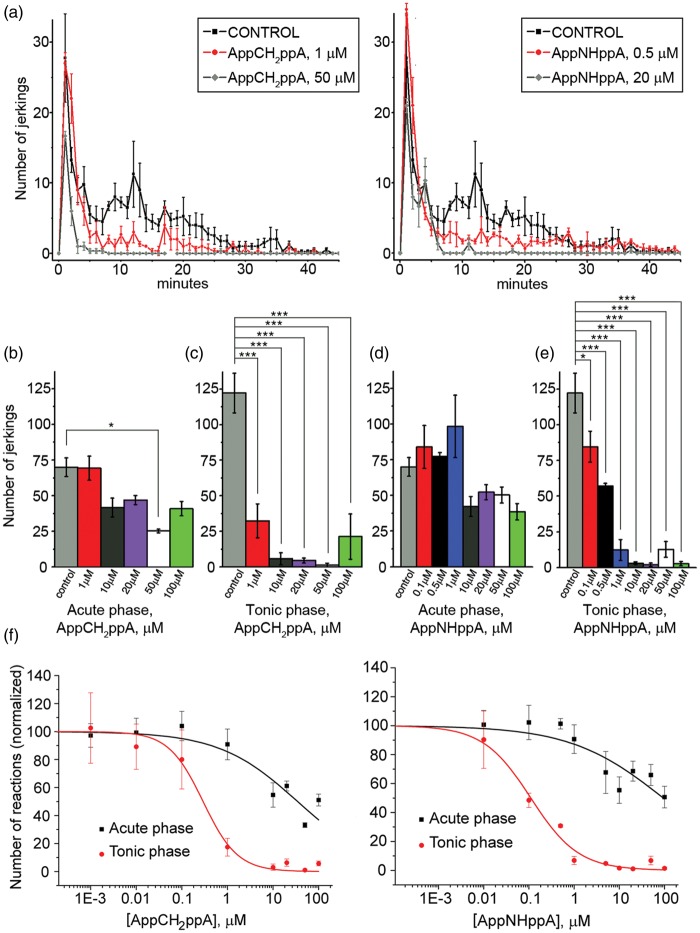
Effects of subcutaneous Ap_4_A analogs on pain responses induced by intraplantar (right hindpaw) formalin injection. Rats were subcutaneously injected with formalin solution alone (0.5%, 50 µl, control) or coinjected with the formalin solution and solutions with AppCH_2_ppA (1 µM–100 µM, 100 µl) or AppNHppA (0.1 µM–100 µM, 100 µl). Age-matched control animals were injected with saline. (a) Time course of the effects of subcutaneous AppCH_2_ppA (left) and AppNHppA (right) on the number of the spontaneous jerkings of the injected paw. (b) Bar graphs of the effects of AppCH_2_ppA (b, c) and AppNHppA (d, e) on the integral number of spontaneous jerkings. Measurements were made during the acute phase (0–6 min) and tonic phase (7–45 min) of the formalin response. (f) Dose-response curves for AppCH_2_ppA (left) and AppNHppA (right) during tonic and acute phases. For each concentration 6–7 rats were used.

**Figure 9.**
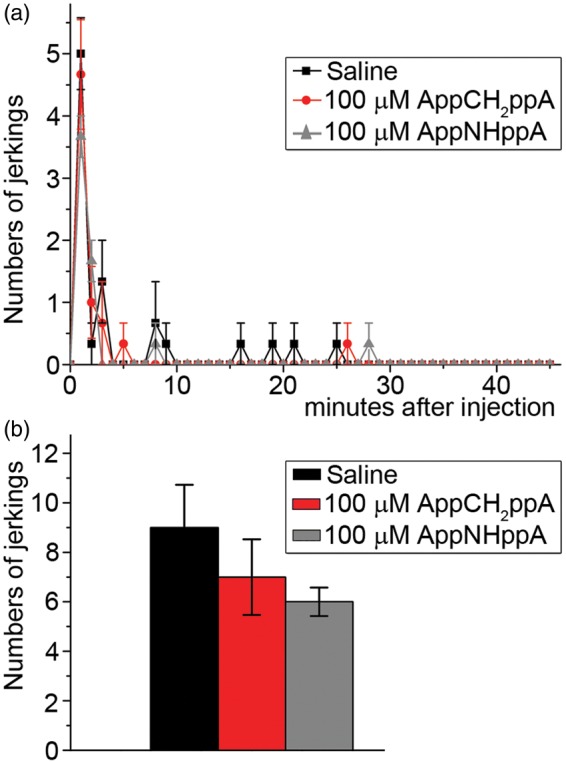
Control injection of 100 μM of AppCH_2_ppA and AppNHppA. (a) Time course of the effects of subcutaneous AppCH_2_ppA and AppNHppA on the number of the spontaneous jerkings of the injected paw. (b) Bar graphs of the effects of AppCH_2_ppA, AppNHppA, and saline on the integral number of spontaneous jerkings. For each compound, three rats were used.

The effects of AppNHppA and AppCH_2_ppA were examined on the behavioral reactions of rats in inflammatory pain models. The injection of diluted formalin into an animal’s hindpaw produces a biphasic nociceptive response consisting of immediate (acute phase) and tonic (inflammatory^17^ phase) components.^55,56^ The first (acute) phase of a formalin response (i.e., 0–5 min after injection) involves a direct effect of formalin on the nociceptive receptors, while changes in animal behavior during the tonic (chronic) phase (i.e., 7–45 min after injection) are caused by hyperalgesia that develops due to the sensitization of nociceptive and spinal neurons^57^ via mechanisms that are triggered by repetitive stimulation during the acute phase.^58^ Coinjection of AppNHppA or AppCH_2_ppA and formalin into the inflamed paw of rats reduced potently the number of nocifensive events triggered by formalin. IC_50_ values were calculated for acute and chronic phases based upon the locally administered concentrations of AppNHppA or AppCH_2_ppA and assuming negligible dilution in tissue postadministration prior to effects on receptors. Accordingly, the IC_50_ value for AppCH_2_ppA in the tonic phase was 0.29 ± 0.05 µM, k_H_ = 1.15 ± 0.17, whilst the IC_50_ value in the acute phase was 34.66 ± 13.21 µM; k_H_ = 0.5 ± 0.14. In comparison, the IC_50_ value for AppNHppA in the tonic phase was 0.11 ± 0.02 µM, k_H_ = 0.82 ± 0.1, while the IC_50_ value in the acute phase was > 100 µM. (Figure 8). Specifically, post AppCH_2_ppA administration, nocifensive behavior was reduced 60 times more effectively in the tonic phase compared with the acute phase of the formalin assay (Figure 8). Calculations based upon the effective concentrations of AppCH_2_ppA and AppNHppA in the formalin model gave respective in vivo IC_50_ values of 0.72 nmol/kg (0.61 µg/kg) and 0.28 nmol/kg (0.23µg/kg) of animal body weight. In control conditions, we observed no sign of nociceptive behavior after injection of Ap_4_A analogs alone in the absence of formalin challenge (Figure 9). Overall, these in vivo IC_50_ values for the tonic phase are in surprisingly good agreement with those IC_50_ values determined using Ap_4_A analogs acting on recombinant rat homomeric P2X3Rs (Figures 2 and 3).

**Figure 10.**
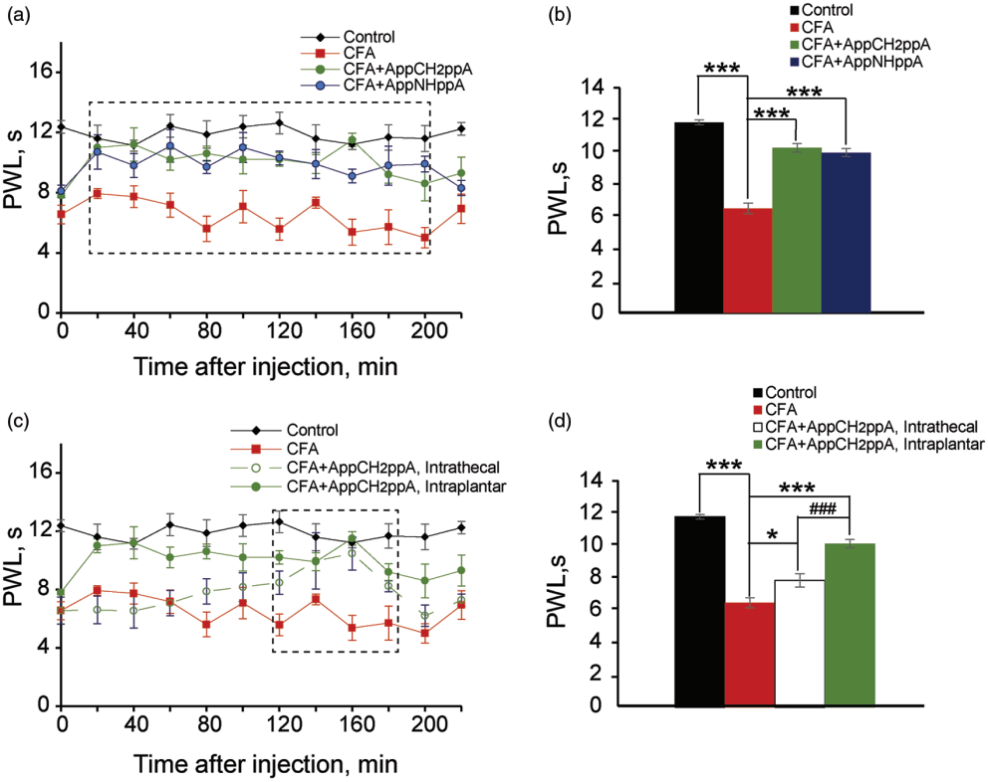
Ap_4_A analogs reduced thermal hyperalgesia induced by complete Freund adjuvant (CFA) (Hargreaves plantar test). (a) Time course of the effects of subcutaneous injection of AppCH_2_ppA (20 µM) and AppNHppA (20 µM) on the paw withdrawal latency (PWL). A dashed box indicates time window, in which at each time point Ap_4_A analogs significantly (*p* < 0.05 for each point) reduced CFA-induced thermal hyperalgesia. (b) Bar graphs illustrating the effects of of AppCH_2_ppA (20 µM) and AppNHppA (20 µM) on PWL. (c) Time course of effects of intraplantar and intrathecal administration of AppCH_2_ppA (20 µM) on PWL. A dashed box indicates a time window, in which at each time point AppCH_2_ppA significantly (*p* < 0.05 for each point) reduced CFA-induced thermal hyperalgesia. (d) Bar graphs illustrating effects of intathecal and intraplantar administration of AppCH_2_ppA (20 µM) on the PWL (* vs. CFA, *p* < 0.05; *** vs. CFA, *p* < 0.001; ### vs. intrathecal, *p* < 0.001; seven rats for control, eight rats for CFA, six rats for intrathecal, and six rats for each compound for intraplantar injection were used).

Subsequently, we tested whether our stable, synthetic Ap_4_A analogs might possess analgesic properties in a model of chronic thermal hyperalgesia induced by the injection of CFA into rat hindpaws. In the Hargreaves plantar test (see Methods for details), an antinociceptive effect was observed during first 200 min following intraplantar injection of AppNHppA and AppCH_2_ppA into the inflamed hindpaw of rats with CFA-induced thermal hyperalgesia (Figure 10(a) and (b)). The effect became potent in 20 min after the injection of AppNHppA or AppCH2ppA and lasted next 180 min (a dashed box in Figure 10(a)). Importantly, intrathecal injection of AppCH_2_ppA (20 µM) induced significantly weaker and delayed antinociceptive effect on CFA-induced thermal hyperalgesia. The effects of intrathecal administration were observed only 120 min post AppCH_2_ppA injection and lasted only 60 min (a dashed box in Figure 10(c)) as compared to 180 min in the case of intraplanar injection. Furthermore, we observed that pain inhibition by intrathecal administration was only 22% compared to 58% inhibition following intraplantar administration (Figure 10(c) and (d)). It is worth noting that Ap_4_A analogs exhibited no obvious effects on thermal hyperalgesia in a sciatic nerve injury model and in a diabetic neuropathy model (data not shown).

## Discussion

Two stable, synthetic Ap_4_A analogs (AppCH_2_ppA and AppNHppA) were studied and found to exhibit remarkably high levels of inhibitory activity on rat and human P2X3Rs that correlates with comparable high levels of antipain activity in the formalin pain model in *vivo*.

### Synthetic Ap_4_A analogs selectively inhibit P2X3Rs

Importantly, P2X3Rs have been implicated in abnormal pain signaling in various states of chronic pain, including inflammatory and cancer-associated pain.^5,22,28,59–63^ Inflammatory mediators such as substance P and bradykinin potentiate currents through ATP-gated channels containing P2X3 subunits.^64^ Recently data suggest that nerve growth factor and protease-activated receptor 2 might trigger sensitization of pain through P2X3R activation.^65–68^ An increased expression of P2X3R has been described at peripheral nerves during painful neoplasic processes.^5,60,62^ Out of several subtypes of the ionotropic P2XRs, P2X3 subunit containing receptors are preferentially expressed in nociceptive sensory neurons^22^ and, therefore, might be therapeutic targets for the treatment of pain.

Several potential strategies might be evoked to counteract enhanced activities of P2X3Rs. Previous studies have shown that P2X3R antagonists or genetic deletion have analgesic effects on inflammatory and neuropathic pain models.^3,9,11^ P2X3Rs are known to exhibit fast onset desensitization in the presence of the agonist followed by an exceptionally slow recovery process.^32,53,69^ P2X3R homo-trimers are also known to be super-sensitive to HAD inhibition by very low concentrations of agonists such as ATP (nM levels).^32,33^ The process of HAD and slow re-sensitization were recently explained as a consequence of the quasi-trapping of receptors in desensitized states caused by fast agonist association but only slow dissociation.^33,53^ HAD is known to be almost absent in homomeric P2X2 and heteromeric P2X2/P2X3Rs.^33^ Consistent with this view, measurements using labeled ATP have demonstrated that ATP dissociation and receptor re-sensitization kinetics correlate closely.^53^ Since P2X3Rs are highly prone to desensitization,^33,70^ this mechanism would seem to present an ideal opportunity to diminish ATP-mediated chronic nociceptive pain, provided that potential analgesics were able to induce HAD without being potent agonists. Importantly, the antinociceptive effect of P2X3 receptor desensitization in vivo has been first shown in the seminal paper by Bland-Ward and Humphrey.^71^ The authors reported that pretreatment with a low dose of α,β-meATP significantly inhibited nociceptive responses induced by α,β-meATP itself; however, this pretreatment was not effective in formalin assay.

Here, we demonstrate that stable, synthetic Ap_4_A analogs (AppCH_2_ppA and AppNHppA) are capable of high-affinity inhibition of rat or human P2X3Rs by stable, use-dependent HAD (but not other P2XRs known to be of significance to nociception) at concentrations that did not generate the macroscopic currents. Critically, these two Ap_4_A analogs (especially AppNHppA) are also found to be only weak agonists of P2X3Rs at higher, nonoverlapping concentrations. Furthermore, this inhibitory effect of our Ap_4_A analogs was almost absent in NG neurons, where α,β-meATP generated mainly slow-type currents mediated by heteromeric P2X2/3Rs. In contrast, the inhibition was strong in neurons that preferentially express homomeric P2X3Rs that are particularly susceptible to desensitization (^47^ and current study). In toto, these results suggest that both synthetic, stable Ap_4_A analogs are able to mediate inhibition by stabilization of the desensitized receptor state. These distinctive properties interlock to support the fact that our stable, synthetic Ap_4_A analogs have characteristics highly appropriate for therapeutic applications directed at the management of chronic pain.

### Potential therapeutic applications of the Ap_4_A analogs

Endogenous Ap*_n_*As are abundant in various tissues including CNS. However (as noted earlier), endogenous Ap*_n_*As are not only potent P2X3R agonists but they are also unstable, which limits their potential utility. However, unlike endogenous Ap*_n_*As, synthetic Ap*_n_*A analogs, prepared with chemical modifications, appear chemically and metabolically stable.^72^ Gratifyingly, the abilities of AppNHppA and AppCH_2_ppA to inhibit P2X3Rs appear to correlate well with analgesic activities test. Indeed a recalculation of the effective concentrations of AppCH_2_ppA and AppNHppA in the formalin model gave respective in vivo IC_50_ values of 0.72 nmol/kg (0.61 µg/kg) and 0.28 nmol/kg (0.23 µg/kg) of animal body weight. Therefore, our compounds are effective antinociceptive at doses that compare well with reported P2X3R antagonists. Clearly, there is a good correlation between Ap_4_A analog P2X3R inhibition and analgesic activities observed in vivo. Accordingly, we would propose that use-dependent HAD of P2X3Rs is the main mechanism contributing to observed inflammatory pain relief mediated by our Ap_4_A analogs.

In support of this proposal, the tonic phase of the formalin test is known to be largely eliminated in P2X3R knock-out mice consistent with a primary role for P2X3R subunits in pain nociception in this pain model.^8,15^ In addition, the selectivity of Ap_4_A analogs for P2X3Rs over P2X2Rs and other P2XRs suggests that the inhibitory actions of our Ap_4_A analogs (especially AppNHppA) should block ATP-gated pain transduction preferentially in TG and DRG neurons as opposed to NG neurons. Future preclinical and clinical studies will help to assess whether Ap_4_A analogs are active as pain killers in TG neuron-related nociceptive pain such as migraine headaches (where the dominating involvement of P2X3Rs is caused by the migraine mediator calcitonin gene-related peptide (CGRP)^66^). Similarly, preclinical and clinical studies will help to assess whether Ap_4_A analogs are active as pain killers in DRG cell-related nociceptive pain as well (such as abdominal visceral and somatic parietal pain, and bone cancer pain).

With respect to future preclinical and clinical evaluations, our experiments demonstrated that local peripheral intraplantar injections of Ap_4_A analogs resulted in antinociceptive effects twofold greater than intrathecal administration (58% vs. 22%) following administration of AppCH_2_ppA. Furthermore the onset of antinociceptive action was much faster in the case of local injection too. These data are consistent with the primary involvement of peripheral P2X3Rs in antinociception rather than spinal P2X3Rs. Therefore, our Ap_4_A analogs have a primary ability to prevent activation of nociceptive the development of pathological plasticity with sensitization in higher structures.

## Conclusion

In summary, in this study we directly show that stable, synthetic analogs of Ap_4_A are weak partial agonists of P2X3Rs that inhibit rat or human recombinant and native rat P2X3Rs at much lower concentrations by HAD that results in stabilization of the desensitized receptor state. Therefore, stable analogs of Ap_4_A represent a new class of potent painkillers for chronic nociceptive pain in in vivo animal models, and potentially in humans. These antinociceptive effects (1) are selective; (2) exhibit use-dependent mechanisms of action; (3) act at the peripheral site where target P2X3Rs are located; and (4) are analogous to endogenous compounds. Thus, we suggest that stable, synthetic Ap_4_A analogs could represent a new highly attractive class of analgesic for the management of chronic inflammatory pain states associated with high activities of nociceptive, ATP-gated receptor mechanisms.
